# Isolated Splenic Infarction: An Atypical Presentation Of Infectious Mononucleosis

**DOI:** 10.7759/cureus.82970

**Published:** 2025-04-25

**Authors:** Muhammad A Akhtar, Thuy H Nguyen, Yasaman Navari, Pradip Chaudhary, Huda Marcus

**Affiliations:** 1 Internal Medicine, Michigan State University, Flint, USA; 2 Internal Medicine, Hurley Medical Center, Flint, USA

**Keywords:** epstein barr virus, hypercoagulability, infectious mononucleosis, rare presentation, splenic infarction

## Abstract

Splenic infarction, being one of the rare and serious complications of infectious mononucleosis, occurs due to splenomegaly and local vascular congestion, sometimes accompanied by transient hypercoagulability. However, it is usually not the presenting complaint. We present the case of a 24-year-old obese male with a past medical history of sleep apnea who presented to our ED with a complaint of moderate to severe left upper quadrant pain. His abdominal pain had continued intermittently for two weeks and progressively worsened, prompting him to come to the hospital. Left upper quadrant tenderness was noted during the physical examination. CT scan of the abdomen and pelvis with contrast revealed moderate splenomegaly and multiple hypodense lesions in the spleen consistent with infarcts. The infectious mononucleosis screen and serological tests for Epstein-Barr virus were positive. However, the patient lacked classic symptoms of infectious mononucleosis. Diagnosis of infectious mononucleosis-associated splenic infarction was made, and the patient was started on conservative management in the hospital. The patient was discharged after five days without complications and was advised to avoid contact sports and strenuous activity for at least four weeks. This case illustrates that splenic infarction can be a presenting symptom in otherwise missed infectious mononucleosis infection and highlights the importance of an elaborate history, examination, and laboratory workup.

## Introduction

Infectious mononucleosis (IM) mainly impacts young people, often causing symptoms such as fever, a sore throat, and enlarged lymph nodes. Other prevalent clinical manifestations consist of splenomegaly, tonsillitis, palatal petechiae, hepatomegaly, and jaundice [1].

The primary pathogen involved is the Epstein-Barr virus (EBV), also known as human herpesvirus 4. EBV is a globally prevalent herpesvirus that spreads through the exchange of bodily fluids, particularly saliva. Infections contracted in childhood are usually asymptomatic, with the highest incidence observed in individuals aged 15-24 [1]. EBV targets B-lymphocytes in the mucosal epithelium of the oropharynx through CD21 receptors. This infection activates both humoral and cellular immune responses, resulting in the spread of the virus throughout the lymphoreticular system and an increase in atypical lymphocytes in the blood. Atypical lymphocytes are primarily CD8-positive cytotoxic T-cells, which target the infected B-lymphocytes [2].

Although EBV is responsible for the majority of infectious mononucleosis cases, around 10% are EBV-negative, typically caused by other pathogens like cytomegalovirus (CMV), human immunodeficiency virus (HIV), toxoplasma, HHV-6, and HHV-7. It is important to note that CMV can lead to a type of mononucleosis that resembles, but is less severe than, the form associated with EBV [3].

Following recovery from infectious mononucleosis, the virus may continue to be excreted in saliva for prolonged periods, sometimes lasting as long as 18 months [4]. The diagnosis of EBV-associated infectious mononucleosis should be considered in young adults presenting with characteristic symptoms. The Monospot test, which identifies heterophile antibodies, is the most frequently used initial test because of its affordability and quick results, despite having lower accuracy. To confirm the diagnosis, EBV-specific antibody testing should be conducted, as it offers 97% sensitivity and 94% specificity [5]. This serological testing is essential, especially because CMV-related mononucleosis infections can result in severe complications for pregnant patients. Additional laboratory findings that may support the diagnosis include an increased absolute lymphocyte count (>4500/microL), atypical lymphocytosis (more than 10% of total lymphocytes), and elevated liver transaminases [6]. Infectious mononucleosis can result in spleen-related complications, including splenomegaly, infarction, and rupture. Among these, splenic infarction is a rare complication and typically occurs after the onset of the classic clinical features of the disease.

## Case presentation

A 24-year-old obese male presented to the emergency department (ED) with moderate to severe left upper quadrant pain. His symptoms of abdominal pain and myalgia started two weeks prior. Myalgia improved; however, his abdominal pain persisted intermittently over the two weeks and progressively worsened. His past medical history was notable only for sleep apnea and exposure to secondhand smoke, with no history of trauma.

Upon arrival in the ED, the patient was afebrile and vitally stable. Physical examination revealed tenderness in the left upper quadrant without other notable findings. Laboratory findings indicated leukocytosis (WBC 16.4) and elevated liver transaminases (alanine transaminase (ALT) 107 U/L, aspartate transferase (AST) 48 U/L). EKG and echocardiogram were unremarkable. Blood cultures returned negative, and serologies for hepatitis, HIV, and other viral infections were also negative. A CT scan of the abdomen and pelvis with contrast demonstrated moderate splenomegaly and multiple hypodense lesions within the spleen, suggestive of infarcts. 

**Figure 1:**
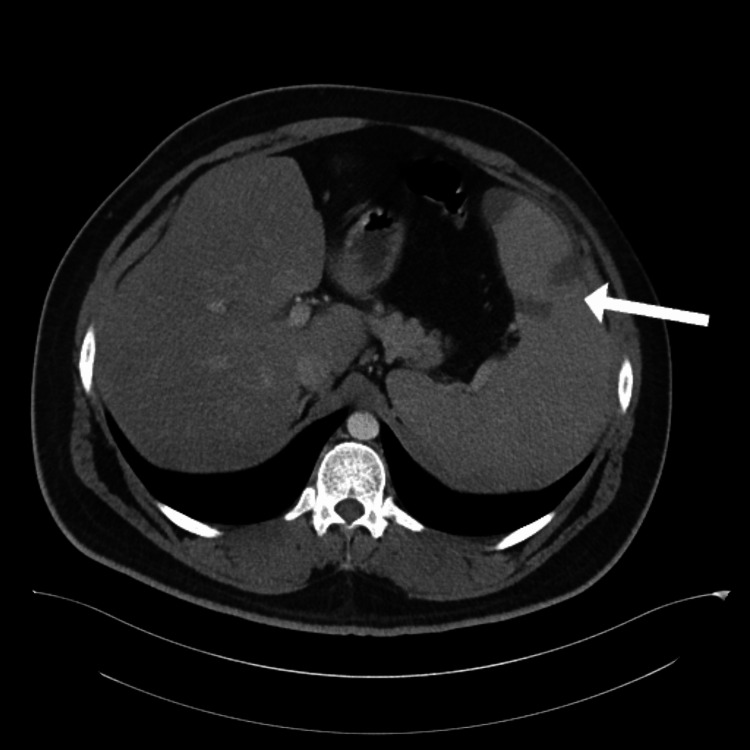
Transverse plane CT scan of abdomen showing multiple infarcts in spleen (white arrow)

**Figure 2:**
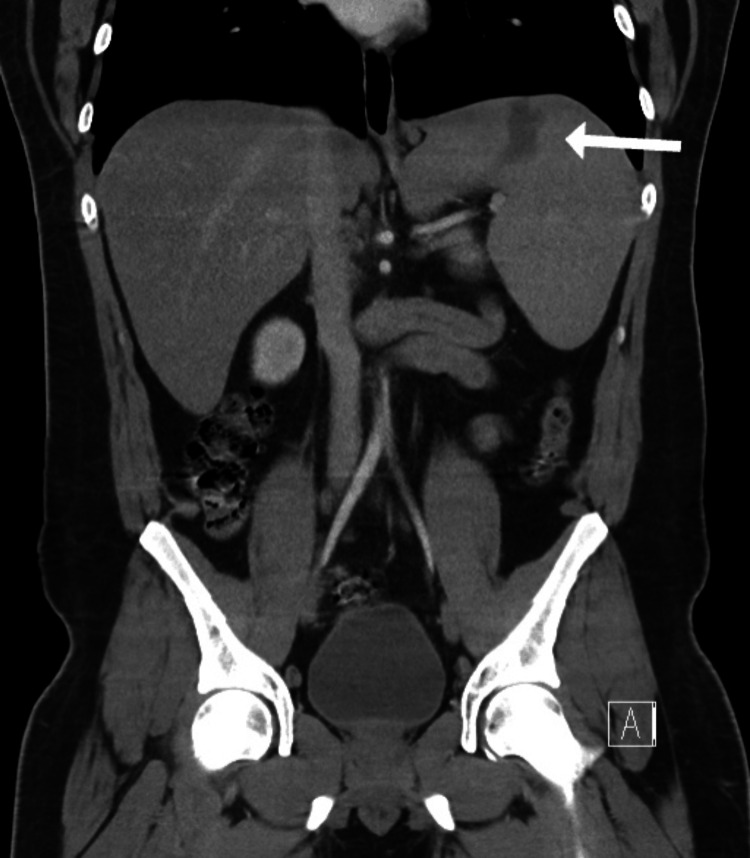
Coronal plane CT scan of abdomen showing splenomegaly and splenic infarction (white arrow)

Given the clinical picture, infectious mononucleosis was considered a differential diagnosis. An infectious mononucleosis screen and serological tests for Epstein-Barr virus (EBV) were conducted, which came back positive.

The hypercoagulability workup showed the presence of lupus anticoagulants, reduced protein C and S activity, and positive IgM cardiolipin antibodies. While EBV infection can trigger the temporary appearance of antiphospholipid antibodies, it is not a direct cause of antiphospholipid syndrome in the majority of cases. To evaluate for any underlying disorders, such as antiphospholipid syndrome, hematology-oncology was consulted in the hospital. The patient also followed up with them in an outpatient setting, and further evaluation confirmed the transient nature of the hypercoagulable state. A Doppler ultrasound of the abdomen excluded arterial thrombosis and thrombosis in the portal, hepatic, and splenic veins. An echocardiogram was performed, which was negative for thrombus or valvular vegetations.

**Figure 3:**
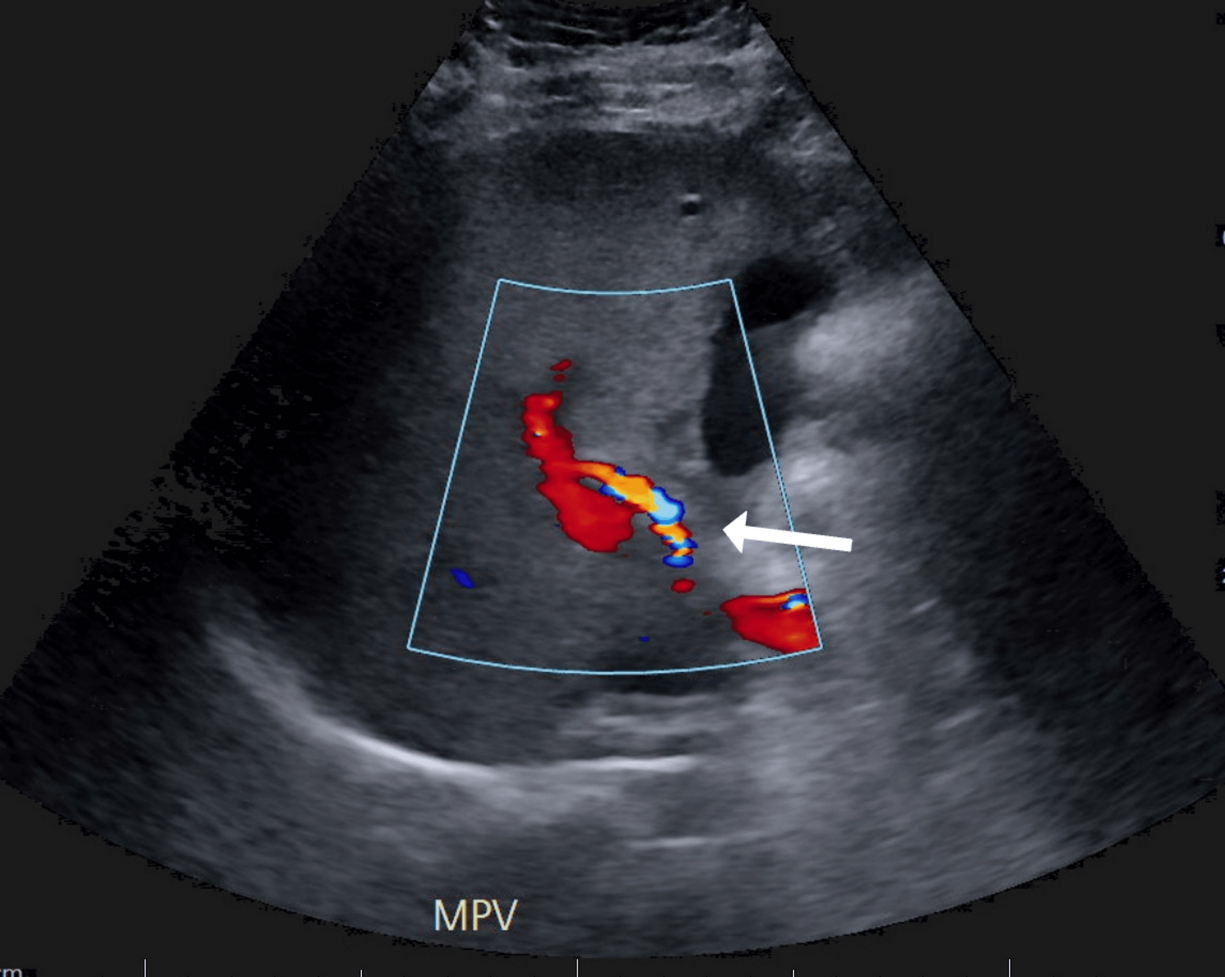
Doppler ultrasound showing blood flow in portal, hepatic and splenic veins (white arrow)

A diagnosis of infectious mononucleosis-associated splenic infarction was established. The patient was managed conservatively in the hospital and was discharged after five days without complications, with instructions to avoid contact sports and strenuous activities for at least four weeks. The patient underwent a repeat CT scan of the abdomen and pelvis as an outpatient eight weeks later, which showed improvement in splenomegaly and a decrease in the hypodense areas of the spleen, consistent with healing infarcts.

**Figure 4:**
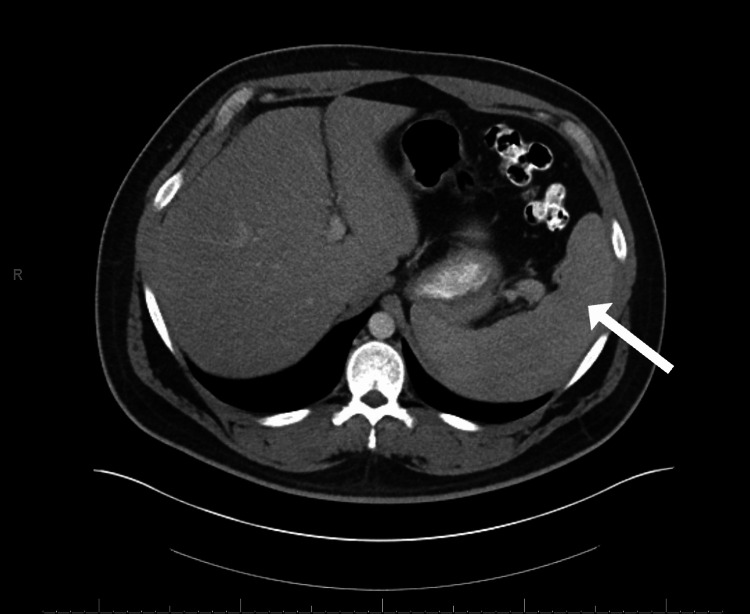
Transverse plane CT scan of abdomen showing improvement in infarcted areas (white arrow)

## Discussion

Splenic infarction, although an uncommon complication of infectious mononucleosis, generally develops after the typical clinical signs of the illness. Most documented cases have reported symptoms such as fever and pharyngitis appearing before abdominal pain. However, a single case in 2022 highlighted abdominal pain as the first symptom of infectious mononucleosis, occurring prior to the onset of fever and pharyngitis [7]. In that case, the patient presented with abdominal pain and splenic infarction, but during his hospital stay, he developed classic features of fever and pharyngitis. In our case report, the patient was a healthy young male without comorbidities who did not exhibit the classic symptoms of infectious mononucleosis. The only reported symptoms were myalgia and abdominal pain.

Infectious mononucleosis presents with various clinical manifestations, and some cases may exhibit milder forms, which can lead to missed diagnoses. It is essential to recognize that infectious mononucleosis can sometimes manifest with just myalgia and pharyngitis, with little to no cervical lymphadenopathy. The hallmark symptoms generally include fever, pharyngitis, tonsillitis, adenopathy, fatigue, palatal petechiae, and splenomegaly. The absence of fatigue and cervical lymphadenopathy reduces the probability of the diagnosis [8]. Hepatomegaly and jaundice may also be present, with posterior cervical and auricular lymph nodes frequently affected. These symptoms typically reach their peak during the first week of the infection. Splenic complications, such as rupture and infarction, are more common in males between the ages of 15 and 30 and usually occur one to three weeks after the onset of initial symptoms. These complications typically present with diffuse abdominal pain, often localized to the left upper quadrant, accompanied by left shoulder pain [1].

Common causes of splenic infarction include malignancies, antiphospholipid syndrome, embolic events, myeloproliferative disorders, sickle cell disease, trauma, and infections such as mononucleosis. While the exact mechanism of splenic infarction in infectious mononucleosis is not fully understood, it may be related to compromised blood flow due to the spleen's hypercellular state or a temporary increase in coagulability [9]. The hypercellularity increases metabolic demand and can lead to localized ischemia. The transient hypercoagulable state can promote thrombosis within the splenic vasculature, leading to infarction. Splenomegaly occurs in approximately 50-60% of cases of infectious mononucleosis, although spleen-related complications are rare. Splenic rupture occurs in approximately one to two per 1000 cases, with a notable incidence in males under 30 [10].

In our literature search, splenic infarction as a complication of infectious mononucleosis was almost invariably preceded by characteristic clinical symptoms of infectious mononucleosis. We present a unique case where splenic infarction in the setting of infectious mononucleosis was not preceded by classic symptoms of infectious mononucleosis.

## Conclusions

Splenic infarction is a rare complication of EBV-associated infectious mononucleosis, with fewer than 35 cases documented in the literature. Our case underscores that milder variants of EBV-associated infectious mononucleosis may not present with typical features, potentially leading to missed diagnoses. It also emphasizes that splenic infarction can occur in otherwise healthy mononucleosis patients without any underlying health issues. This case illustrates that splenic infarction can serve as an initial symptom of an undiagnosed infectious mononucleosis infection. It highlights the importance of thorough history-taking, examination, and laboratory evaluation. Therefore, infectious mononucleosis should be considered in the differential diagnosis for patients presenting with splenic infarction, even in the absence of classic symptoms and in otherwise healthy individuals.
